# Black tea, green tea and risk of breast cancer: an update

**DOI:** 10.1186/2193-1801-2-240

**Published:** 2013-05-24

**Authors:** Yili Wu, Dongfeng Zhang, Shan Kang

**Affiliations:** Department of Epidemiology and Health Statistics, The Medical College of Qingdao University, Dongzhou Road, No.38, Shandong, Qingdao 266021 P.R. China

**Keywords:** Black tea, Green tea, Breast cancer, Dose-response analysis

## Abstract

Previous meta-analysis indicated conflicting results in case–control versus cohort studies on the association of green tea with breast cancer risk, and conflicting results were also found in case–control versus cohort studies in another meta-analysis on the association of black tea with breast cancer risk. Many studies were published after the previous meta-analysis. Besides, the dose-response relationship of tea consumption with breast cancer risk is unclear. Thus the association of tea consumption with breast cancer risk was assessed incorporating new publications. Summary relative risk (RR) for highest versus lowest level of tea consumption was calculated based on fixed or random effect models. Dose-response relationship was assessed by restricted cubic spline model and multivariate random-effect meta-regression. The combined results from 9 studies suggested no significant association between green tea consumption and breast cancer risk (RR = 0.82, 95% CI = 0.64-1.04). No significant association was found among cohort studies and case-control studies after sensitivity analysis, respectively. A linear but not significant dose-response association was found between green tea consumption and breast cancer risk. The combined results from 25 studies demonstrated no significant association between black tea consumption and breast cancer risk (RR = 0.98, 95% CI = 0.93-1.03), and no significant association was found in subgroup analysis. A linear but not significant dose-response association was found between black tea consumption and breast cancer risk. Based on the current evidence, black tea and green tea might not contribute significantly to breast cancer risk, respectively.

**Electronic supplementary material:**

The online version of this article (doi:10.1186/2193-1801-2-240) contains supplementary material, which is available to authorized users.

## To the editor

The most recent meta-analysis by Ogunleye *et al.* (Ogunleye et al. 2010) included 7 (2 cohort and 5 case-control) studies of green tea and breast cancer that were published as of December 2008. An inverse association between green tea and breast cancer risk was reported from case-control studies [compared to the lowest quantile, the relative risk (RR) for the highest quantile of green tea is 0.81, 95% CI = 0.75-0.88], while no association was observed from cohort studies (compared to the lowest quantile, the RR for the highest quantile of green tea is 0.85, 95% CI = 0.65-1.22), and the authors concluded that the association between green tea consumption and breast cancer risk remains unclear. Meanwhile, Zhou *et al.* (Zhou et al. 2011) suggested that a dose-response analysis should be performed to assess the association between green tea and breast cancer risk. In another meta-analysis on black tea and breast cancer risk, Sun *et al.*(Sun et al. 2006) included 13 (5 cohort and 8 case-control) studies that were published as of August 2004. A moderate positive association between black tea consumption and risk of breast cancer was observed in cohort studies (compared to the lowest quantile, the RR for the highest quantile of black tea is 1.15, 95% CI = 1.02-1.31) whereas a minor inverse association was observed from the case-control studies (compared to the lowest quantile, the RR for the highest quantile of black tea is 0.91, 95% CI = 0.84-0.98). Following the meta-analyses by Ogunleye *et al.* (Ogunleye et al. 2010), results were published from 2 prospective cohort studies (Iwasaki et al. 2010; Dai et al. 2010) on the association of green tea with risk of breast cancer. And since the meta-analysis by Sun *et al.*(Sun et al. 2006), results were published from 9 prospective cohort studies (Harris et al. 2012; Fagherazzi et al. 2011; Boggs et al. 2010; Bhoo Pathy et al. 2010; Larsson et al. 2009; Ishitani et al. 2008; Ganmaa et al. 2008; Hirvonen et al. 2006; Adebamowo et al. 2005) and 3 case-control studies (Yuan et al. 2005; Kumar et al. 2009; Baker et al. 2006) on the association of black tea with breast cancer risk. Besides, we also would like to draw attention to the dose-response analysis, because categories of tea consumption differed between studies, which might complicate the interpretation of the pooled results across study populations with different categories. In this respect, a dose–response meta-analysis with restricted cubic spline functions provides a solution to the problem (Desquilbet & Mariotti 2010), from which a summary risk estimate can be derived for a standardized increase and specific exposure values for tea consumption.

We performed a literature search to October 2012 using the databases of Pubmed, ISI Web of Knowledge, China Biology Medical literature database and Google Scholar with the key words tea consumption combined with breast cancer. Furthermore, the reference lists of retrieved articles were scrutinized to identify additional relevant studies. If data were duplicated in more than 1 study, we included the study with the largest number of cases. RR estimates with corresponding 95% CI for the highest vs. lowest categories of tea consumption were extracted. For dose-response analysis, the number of cases and participants (person-years), and RR (95% CI) for each category of tea consumption were also extracted. The median or mean level of tea consumption for each category was assigned to corresponding RR for every study. If the upper boundary of the highest category was not provided, we assumed that the boundary had the same amplitude as the adjacent category. We extracted the RR that reflected the greatest degree of control for potential confounders.

Pooled measure was calculated as the inverse variance-weighted mean of the logarithm of RR with 95% CI to assess the strength of association between tea consumption and breast cancer risk. The *I*^*2*^ of Higgins and Thompson was used to assess heterogeneity (*I*^*2*^ values of 0, 25%, 50%, and 75% represents no, low, moderate, and high heterogeneity (Higgins et al. 2003), respectively). The fixed effect model was used as the pooling method if moderate or lower heterogeneity (*I*^*2*^ < 50%) was found, otherwise, the random effect model was adopted (*I*^*2*^ ≥ 50%). Besides, combining studies regardless of the between-study heterogeneity had been widely criticized (Lau et al. 1998), and hierarchical systems for grading evidence stated that the results of studies must be consistent or homogeneous to obtain the highest grading (Harbour & Miller 2001). Thus, sensitivity analysis was also carried out using the method by Patsopoulos *et al.* (Patsopoulos et al. 2008) with *I*^*2*^ > 50% as the criteria to reduce between-study heterogeneity. Publication bias was detected using Egger’s linear regression test (Egger et al. 1997).

A two-stage random-effects dose–response meta-analysis (Orsini et al. 2012) was performed to compute the trend from the correlated log RR estimates across levels of tea consumption taking into account the between-study heterogeneity. Briefly, a restricted cubic spline model, with 3 knots at the 25th, 50th and 75th percentiles (Harrell et al. 1988) of the levels of tea consumption was estimated using generalized least square regression taking into account the correlation within each set of published RRs (Orsini & Bellocco 2006). Then multivariate random-effects meta-analysis was used to combine the study-specific estimates using restricted maximum likelihood method (Jackson et al. 2010). A *P* value for nonlinearity was calculated by testing the null hypothesis that the coefficient of the second spline is equal to 0. If tea consumption was indicated by gram of tea leaves or tea beverage, we rescaled tea consumption to the number of cups per day assuming 2.5 g tea leaves or 150 g tea beverage as approximately equivalent to one cup (Tang et al. 2009). All statistical analyses were performed with Stata software, version 12 (Stata Corp, College Station, Texas). P < .05 was considered statistically significant.

**Figure 1:**
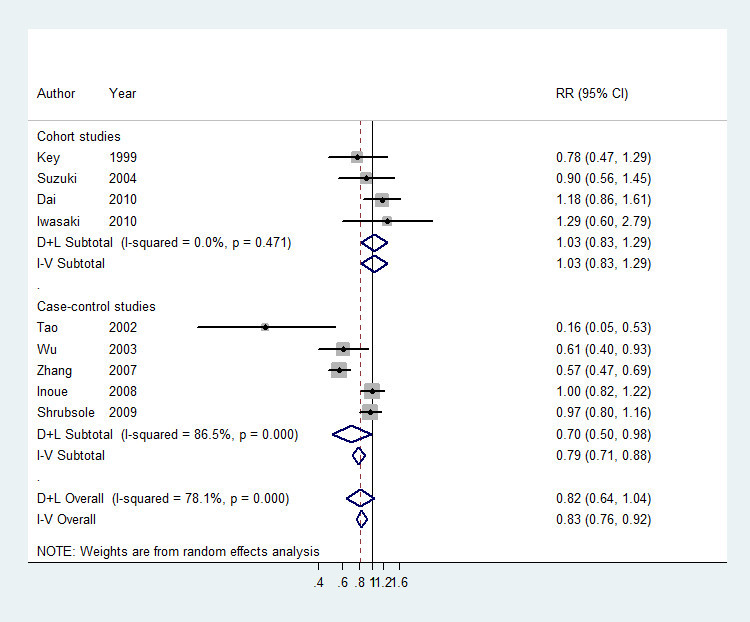
**The multivariate-adjusted risk of breast cancer for the highest vs. lowest categories of green tea consumption.** D + L denotes random effect model, I-V denotes fixed effect model.

**Figure 2:**
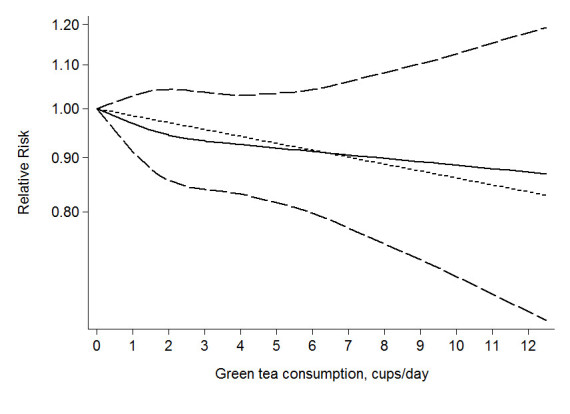
**The dose-response analysis between green tea consumption and breast cancer risk.** The solid line and the long dash line represent the estimated relative risk and its 95% confidence interval. Short dash line represents the linear relationship.

For green tea, data from 9 studies (Iwasaki et al. 2010; Dai et al. 2010; Shrubsole et al. 2009; Inoue et al. 2008; Zhang et al. 2007; Suzuki et al. 2004; Wu et al. 2003; Key et al. 1999; Tao et al. 2002) were used. Compared to the lowest quantile, the RR of breast cancer for the highest quantile of green tea was 0.82 (0.64-1.04), and high between-study heterogeneity was found (*I*^*2*^ = 78.1%) (Figure 1). After sensitivity analysis with *I*^*2*^ > 50% as the criteria, the association was still not significant (RR = 0.96, 95% CI = 0.86-1.08). No significant association was found among cohort studies (RR = 1.03, 95% CI = 0.83-1.29, *I*^*2*^ = 0.00%). A marginally significant association was found among case-control studies (RR = 0.70, 95% CI = 0.50-0.98, *I*^*2*^ = 86.5%), however, the association was not significant after sensitivity analysis (RR = 0.98, 95% CI = 0.86-1.13, *I*^*2*^ = 0.00%). Data from 7 studies (Iwasaki et al. 2010;Dai et al. 2010; Shrubsole et al. 2009; Zhang et al. 2007; Suzuki et al. 2004; Wu et al. 2003; Key et al. 1999) were used for dose-response analysis. A linear (*P* = 0.55) but not significant dose-response association was found between green tea consumption and breast cancer risk (Figure 2), and the risk of breast cancer decreased by 3% (RR = 0.97, 95% CI = 0.90-1.04, *P* = 0.39) for every 2 cups/day increment in green tea consumption. The RR (95% CI) of breast cancer was 0.97 (0.91-1.03), 0.94 (0.86-1.04), 0.93 (0.84-1.04), 0.93 (0.83-1.03), 0.92 (0.81-1.04) and 0.91 (0.79-1.04) for 1, 2, 3, 4, 5 and 6 cups/day of green tea consumption. No publication bias was detected (*P* = 0.68).

**Figure 3:**
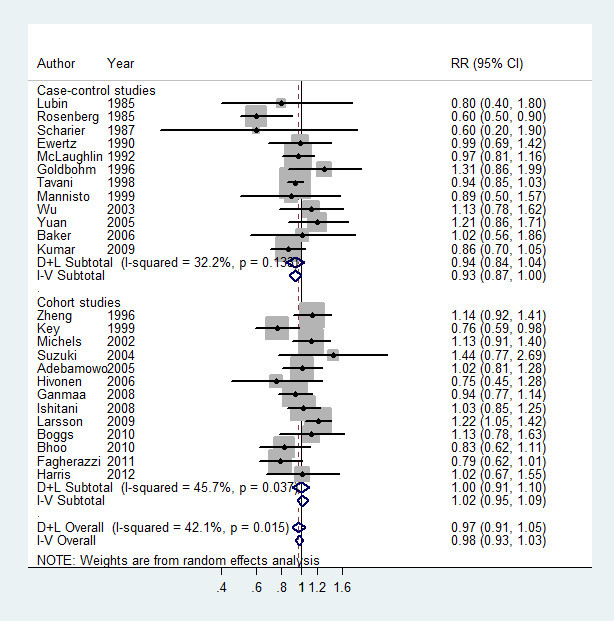
**The multivariate-adjusted risk of breast cancer for the highest vs. lowest categories of black tea consumption.** D + L denotes random effect model, I-V denotes fixed effect model.

**Figure 4:**
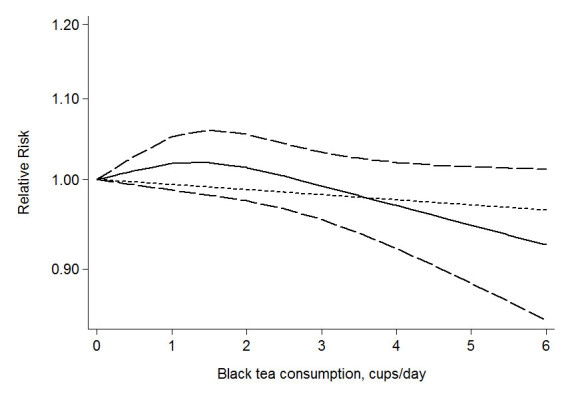
**The dose-response analysis between black tea consumption and breast cancer risk.** The solid line and the long dash line represent the estimated relative risk and its 95% confidence interval. Short dash line represents the linear relationship.

For black tea, data from 25 studies (Harris et al. 2012; Fagherazzi et al. 2011; Boggs et al. 2010; Bhoo Pathy et al. 2010; Larsson et al. 2009; Ishitani et al. 2008; Ganmaa et al. 2008; Hirvonen et al. 2006; Adebamowo et al. 2005; Yuan et al. 2005; Kumar et al. 2009; Baker et al. 2006; Suzuki et al. 2004; Wu et al. 2003;Key et al. 1999; Michels et al. 2002; Zheng et al. 1996; Goldbohm et al. 1996; Mannisto et al. 1999; McLaughlin et al. 1992; Ewertz & Gill 1990; Schairer et al. 1987; Lubin et al. 1985; Tavani et al. 1998; Rosenberg et al. 1985) were used. Compared to the lowest quantile, the RR of breast cancer for the highest quantile of black tea was 0.98, 95% CI = 0.93-1.03, *I*^*2*^ = 42.1%) (Figure 3). The association was also not significant in subgroups by study design categorized as cohort studies (RR = 1.02, 95% CI = 0.95-1.09, *I*^*2*^ = 45.7%) and case-control studies (RR = 0.94, 0.87-1.00, *I*^*2*^ = 32.2%), menopausal status categorized as premenopausal status (RR = 0.92, 95% CI = 0.77-1.08, *I*^*2*^ = 15.6%) and postmenopausal status (RR = 1.07, 95% CI = 0.96-1.21, *I*^*2*^ = 0.00%), estrogen receptor (ER) and progesterone receptor (PR) status (negative: -; positive: +) categorized as ER+/PR + (RR = 1.03, 95% CI = 0.80-1.34, *I*^*2*^ = 63.9%) and ER-/PR- (RR = 0.84, 95%CI = 0.68-1.03, *I*^*2*^ = 0.00%), as well as body mass index (BMI) categorized as BMI < 25 kg/m^2^ (RR = 0.98, 95%CI = 0.81-1.18, *I*^*2*^ = 51.2%) and BMI > 25 kg/m^2^ (RR = 1.02, 95% CI = 0.84-1.24, *I*^*2*^ = 0.00%). After sensitivity analysis with *I*^*2*^ > 50% as the criteria, the association was still not significant for ER+/PR + breast cancer (RR = 0.93, 95% CI = 0.82-1.05, *I*^*2*^ = 0.00%), and no significant association was found among subjects with BMI < 25 kg/m^2^ (RR = 1.07, 95% CI = 0.86-1.33, *I*^*2*^ = 0.00%). Data from 19 studies (Harris et al. 2012;Fagherazzi et al. 2011; Boggs et al. 2010; Bhoo Pathy et al. 2010; Larsson et al. 2009; Ganmaa et al. 2008; Hirvonen et al. 2006; Adebamowo et al. 2005; Kumar et al. 2009; Baker et al. 2006; Wu et al. 2003; Key et al. 1999; Michels et al. 2002; Zheng et al. 1996; Goldbohm et al. 1996; Ewertz & Gill 1990; Schairer et al. 1987; Lubin et al. 1985; Rosenberg et al. 1985) were used for dose-response analysis. A linear (*P* = 0.09) but not significant dose-response association was found between black tea consumption and breast cancer risk (Figure 4), and the risk of breast cancer decreased by 1% (RR = 0.99, 95% CI = 0.96-1.03, *P* = 0.68) for every 2 cups/day increment in black tea consumption. The RR (95% CI) of breast cancer was 1.02 (0.99-1.05), 1.01 (0.98-1.05), 0.99 (0.95-1.03), 0.97 (0.92-1.02), 0.95 (0.89-1.01) and 0.93 (0.85-1.01) for 1, 2, 3, 4, 5 and 6 cups/day of black tea consumption. No publication bias was detected (*P* = 0.79).

Overall, this analysis suggested that black tea and green tea might not contribute significantly to breast cancer risk based on the current evidence, respectively. However, further researches deserve to address the possible interaction effects between tea and other dietary/genetic cofactors.

## Authors’ original submitted files for images

Below are the links to the authors’ original submitted files for images. Authors’ original file for figure 1 Authors’ original file for figure 2 Authors’ original file for figure 3 Authors’ original file for figure 4
