# Supplementary figures and images for: Black tea, green tea and risk of breast cancer: an update
Source: Springerplus. 2013 May 24;2(1):240. doi: 10.1186/2193-1801-2-240 (PMC3671100; doi:10.1186/2193-1801-2-240)

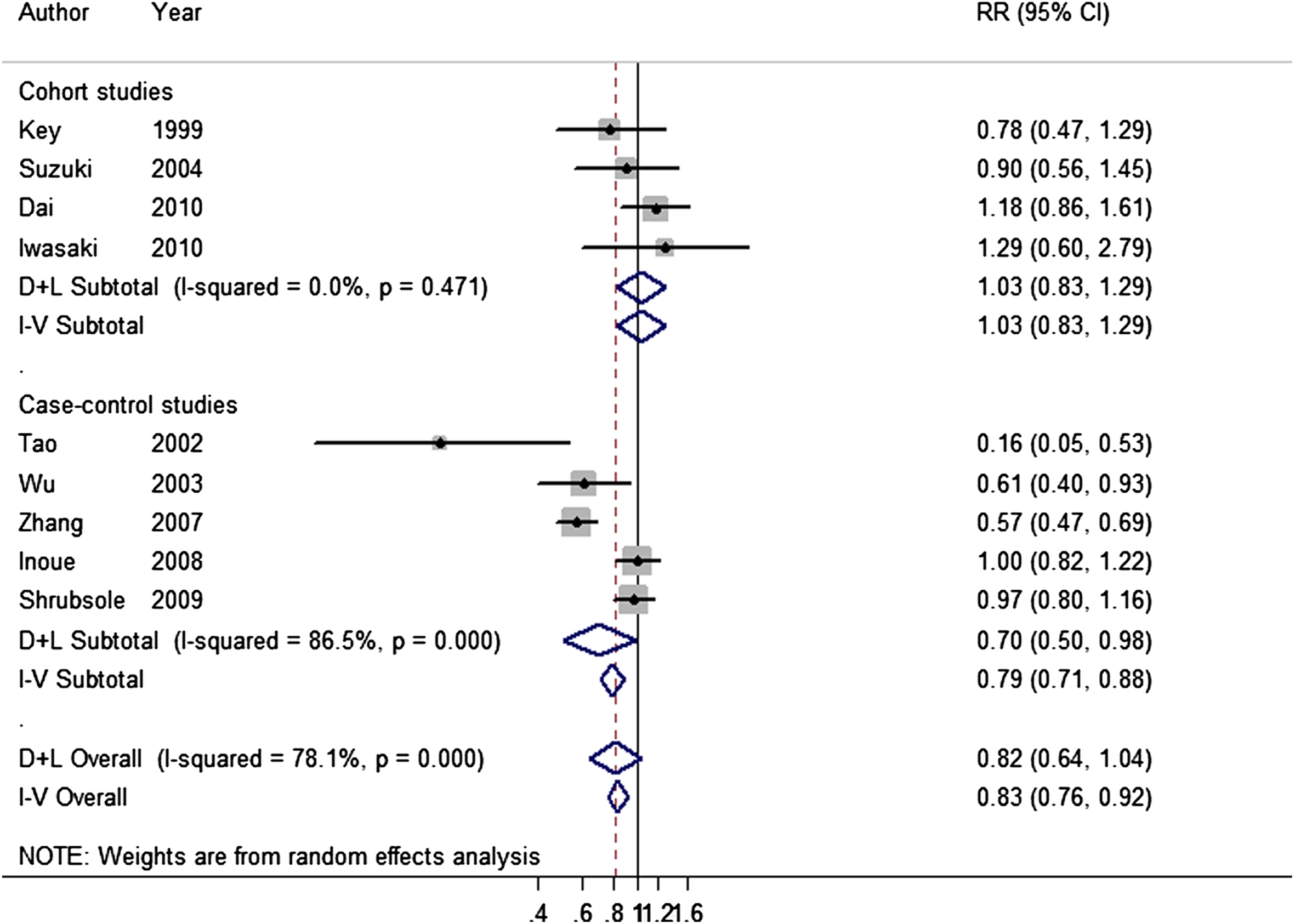

Supplement: Supplementary file 1 — Authors’ original file for figure 1 [file 40064_2013_305_MOESM1_ESM.tiff]

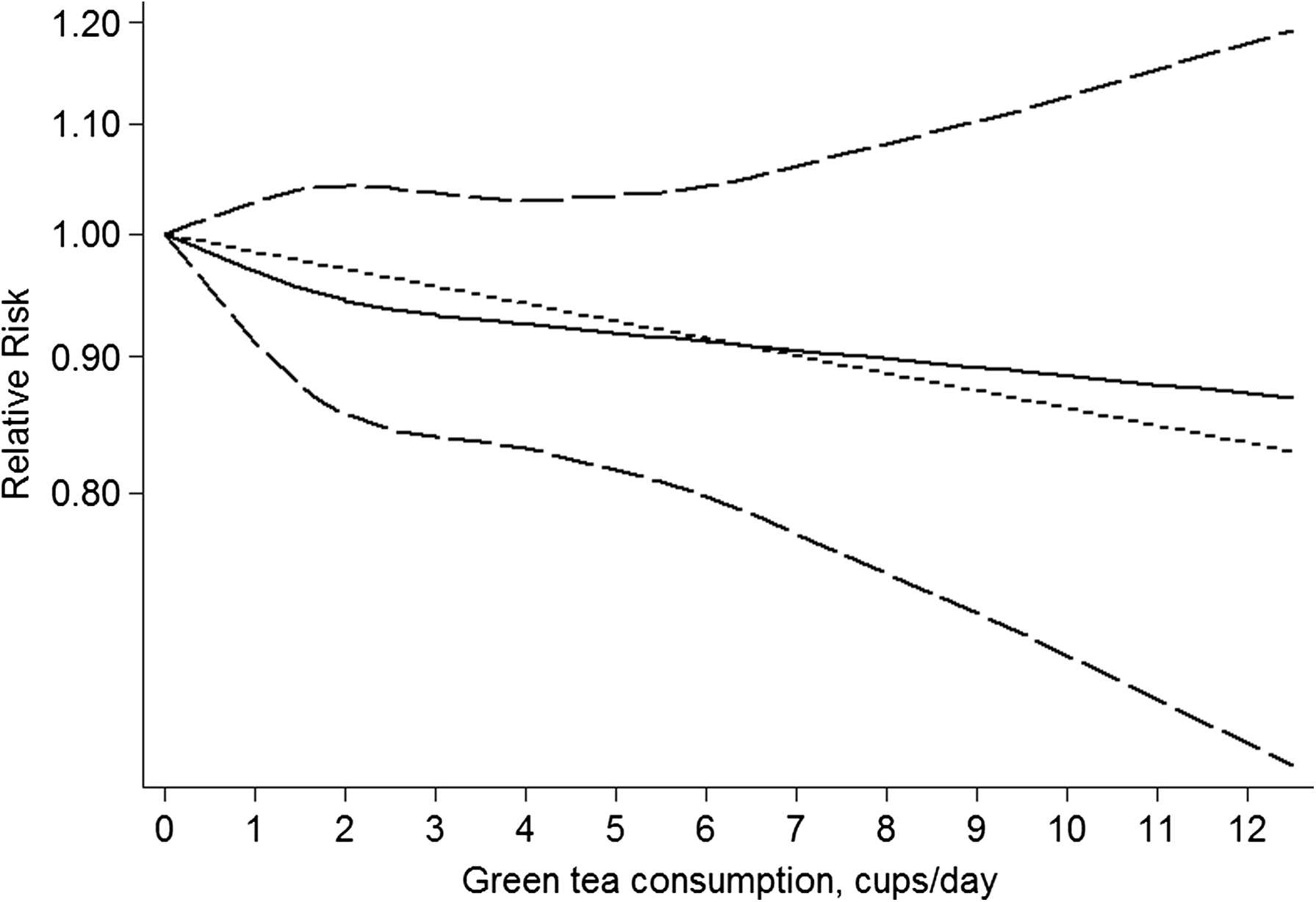

Supplement: Supplementary file 2 — Authors’ original file for figure 2 [file 40064_2013_305_MOESM2_ESM.tiff]

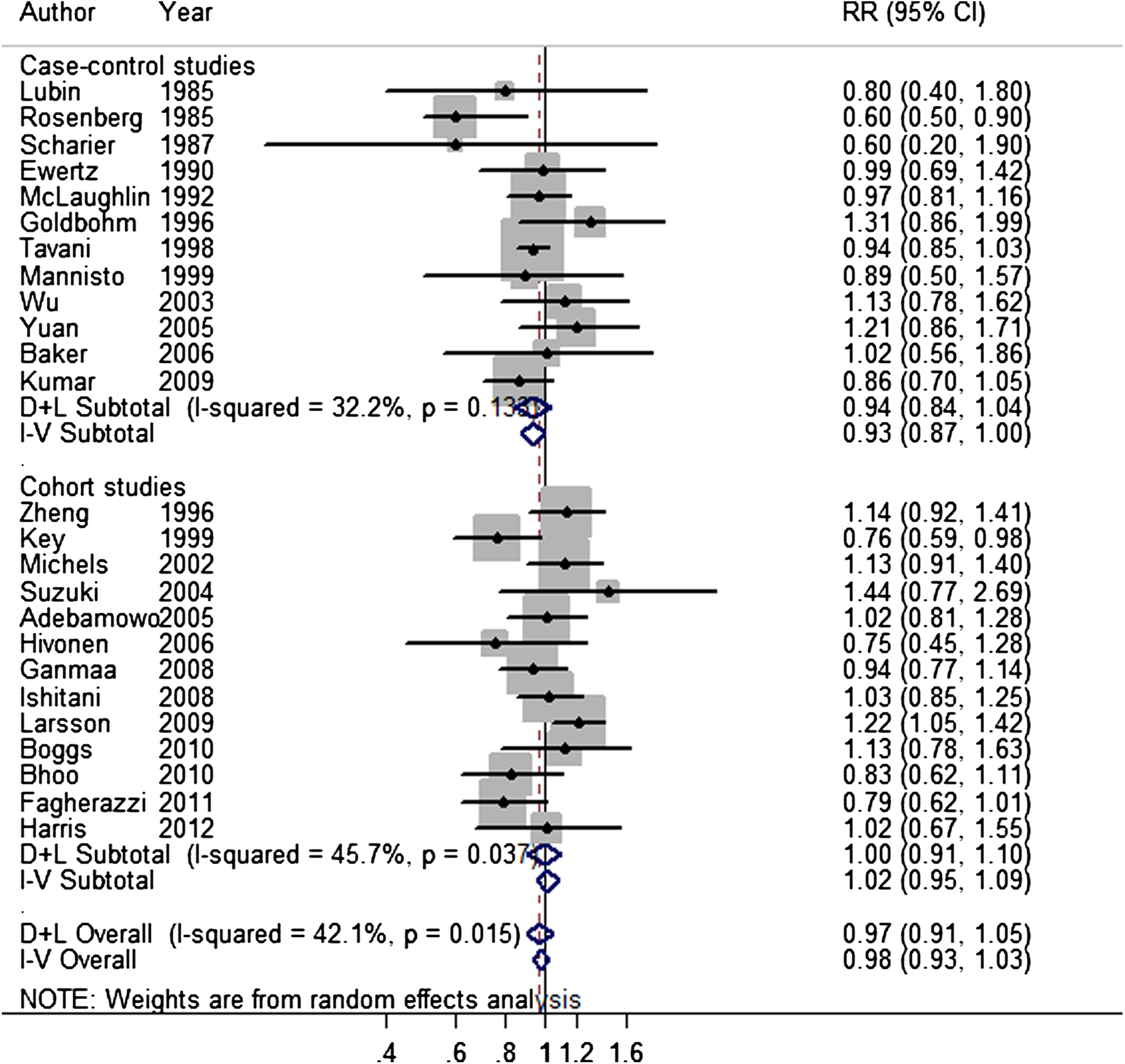

Supplement: Supplementary file 3 — Authors’ original file for figure 3 [file 40064_2013_305_MOESM3_ESM.tiff]

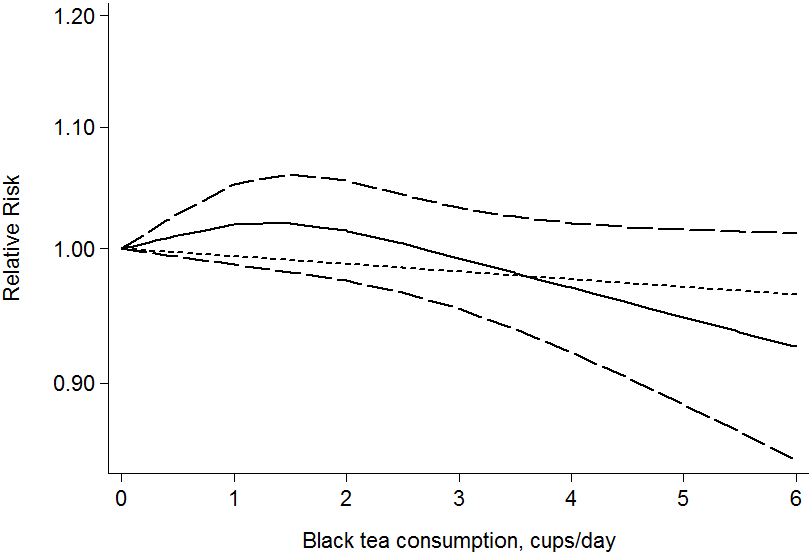

Supplement: Supplementary file 4 — Authors’ original file for figure 4 [file 40064_2013_305_MOESM4_ESM.tiff]
